# Antimicrobial Activity of Five Essential Oils against Bacteria and Fungi Responsible for Urinary Tract Infections

**DOI:** 10.3390/molecules23071668

**Published:** 2018-07-09

**Authors:** Valentina Virginia Ebani, Simona Nardoni, Fabrizio Bertelloni, Luisa Pistelli, Francesca Mancianti

**Affiliations:** 1 Department of Veterinary Science, University of Pisa, Viale delle Piagge 2, 56124 Pisa, Italy; simona.nardoni@unipi.it (S.N.); fabrizio.bertelloni@vet.unipi.it (F.B.); francesca.mancianti@unipi.it (F.M.); 2 Centro Interdipartimentale di Ricerca “Nutraceutica e Alimentazione per la Salute”, University of Pisa, via del Borghetto 80, 56124 Pisa, Italy; luisa.pistelli@unipi.it; 3 Department of Pharmacy, University of Pisa, via Bonanno 6, 56126 Pisa, Italy

**Keywords:** *Escherichia coli*, *Enterococcus*, *Candida*, antibiotic resistance, antimycotic resistance, essential oil

## Abstract

Urinary tract infections are frequently encountered in small animal practice. *Escherichia coli* and *Enterococcus* spp. are the most common agents associated to these infections, even though other bacteria and yeasts, such as *Candida albicans* and *Candida famata,* may be involved. In view of the increasing problem of the multi-drug resistance, the aim of this study was to investigate the antimicrobial activity of essential oils obtained from star anise (*Illicium verum* Hook.f.), basil (*Ocimum basilicum* L.), origanum (*Origanum vulgare* L.), clary sage (*Salvia sclarea* L.) and thymus (*Thymus vulgaris* L.) against multidrug-resistant strains of *Escherichia coli*, *Enterococcus* spp., *Candida albicans* and *Candida famata* previously isolated from dogs and cats with urinary tract infections. Enterococci were resistant to *Illicium verum* and *Salvia sclarea*, such as *Candida* to *Salvia sclarea*. *Thymus vulgaris* and *Origanum vulgare* essential oils showed the best activity against all the tested pathogens, so they could be proposed for the formulation of external and/or intravesical washes in small animals.

## 1. Introduction

Infections of the urinary tract are frequent and sometimes can induce severe threat both in human and veterinary medicine, mostly affecting dogs and cats. Urinary tract infections (UTI) may be localized to the upper tract (kidney and adjacent ureter) or the lower tract (bladder and adjacent urethra) and more than one organ is often involved [1]. These infections are usually caused by bacteria, mainly those of the intestinal microflora. *Escherichia coli* and *Enterococcus* spp. are the most frequent agents encountered in UTI cases [2,3,4]. Even though infections by haematogenous route are possible, bacteria usually colonize the genito-urinary tract by ascendant route. In view of the anatomic structure, females are more prone to UTI than males [1]. 

Yeasts belonging to *Candida* genus, in particular *Candida albicans*, are reported as responsible for fungal infections of the lower urinary tract, both in dogs and cats [5,6]. The organism is a commensal of digestive and genito-urinary tract of both healthy people and animals [7]. It can also act as opportunistic pathogen of skin and mucosae both in receptive animals and in immunocompromised patients [8]. Conversely, other *Candida* species are ubiquitous yeasts and can provoke UTI, when carried by the intestinal content or by environmental contamination. The most severe threat of UTI is the multi-drug resistance of etiological agents that often negatively affects healing.

Enterococci are of severe concern for their intrinsic antibiotic resistance, particularly to cephalosporins and aminoglycosides, or acquired resistance to many other antimicrobials [9]. *E. coli* antibiotic resistance is an increasing problem concerning several antibiotic classes, including the new antimicrobial agents introduced into clinical medicine [10]. Both Enterococci and *E. coli* present in the gastrointestinal habitat may acquire antimicrobial resistance genes from other commensal organisms, transferring them to more pathogenic bacteria [11]. The epidemiology of *Candida* infections has strongly changed in human patients [12], showing an increase of incidence of non-*albicans* species*. Candida* spp. are characterized by marked differences in their antifungal susceptibility pattern [13]. Particularly, *C. albicans* strains are frequently resistant to azoles [14,15]). In veterinary medicine information is scanty, furthermore the choice of molecules allowed for UTI treatment is very limited. Moreover, the majority of antimycotic drugs administered to human patients represent an off-label drug use. 

The spread of drug-resistant pathogens requires novel therapies, so in a view of a natural approach, the use of essential oils (EOs) to treat urogenital infections has been proposed in human medicine [16], whereas no scientific data about their possible employment in animals are available. EOs are secondary plant metabolites, obtained by steam or dry distillation or by means of a mechanical treatment from one single species [17]. They are volatile substances, with odorous impact, characterized by different degrees of antimicrobial activity, in relation to several factors. This property is strictly related to the pathogen; moreover, it depends on the original plant species, climate conditions, cultivation methods or harvesting areas, EOs preparation method and EOs composition [17]. 

Aim of the present study was to investigate the antimicrobial activity of EOs obtained from star anise (*Illicium verum* Hook.f.), basil (*Ocimum basilicum* L.), origanum (*Origanum vulgare* L.), clary sage (*Salvia sclarea* L.) and thymus (*Thymus vulgaris* L.) against multidrug-resistant strains of *E. coli*, *Enterococcus* spp., *C. albicans* and against isolates of *C. famata* characterized by high MIC values for conventional antimycotic drugs, previously isolated from dogs and cats with UTIs.

## 2. Results

### 2.1. Essential oils Analysis

The chemical composition of the tested EOs is reported in Table 1. All the five oils were rich in monoterpenes. In detail, the main terpenes identified in *O. vulgare* and *T. vulgaris* EOs were carvacrol (65.9%) and thymol (52.6%), respectively, followed by *p*-cymene (15.3%) only in *T. vulgaris*. The main compounds of *S. sclarea* and *O. basilicum* were linalyl acetate (54.7%) and linalool (46.0%), respectively. *I. verum* EO was mainly composed by the phenylpropanoid anethol (89.8%). 

### 2.2. Antibacterial Activity

#### Agar Disc Diffusion Method

The results of the agar disc diffusion method testing *E. coli* and *Enterococcus* isolates against 21 antibiotics are summarized in Table 2.

All the strains resulted multi-resistant, even though with different resistance patterns.

High percentages of no-sensitive (resistant or intermediate) strains against several antibiotics were detected, both among the tested *E. coli* and *Enterococcus* spp. isolates (Table 3). 

Table 4 reports the minimum inhibitory concentration (MIC) values, expressed both as percentage and as mg/mL, testing *E. coli* and *Enterococcus* strains against the five selected EOs. *I. verum* and *S. sclarea* EOs did not show any antibacterial activity against *Enterococcus* isolates, whereas they induced moderate growth inhibition versus some *E. coli* strains. Better results have been obtained by the remaining EOs, mainly *O. vulgare* and *T. vulgaris* oils. Results obtained by agar disk diffusion method and broth microdilution test were comparable. No growth inhibition was observed with the negative control.

### 2.3. Antimycotic Activity

Selected yeasts showed different patterns of resistance to conventional antimycotic drugs. In detail, all *C. albicans* isolates were resistant to voriconazole and to itraconazole, 9/12 to fluconazole, while MICs yielded from *C. famata* were high in 3 cases out of 4 for both fluconazole and itraconazole, and in 1/4 case for voriconazole. Caspofungin resulted active for all yeasts isolates.

Selected EOs showed different efficacy against tested yeasts. *T. vulgaris* EO yielded the lowest overall MICs and was effective versus all *C. famata* and versus 11/12 *C. albicans* isolates with MICs lower than 1 mg/mL. *O. vulgare* and *O. basilicum* appeared less active*,* even if the lowest MIC among all tested EOs (0.01%) was obtained by *O. vulgare* versus a strain of *C. albicans*. Conversely, *S. sclarea* was ineffective versus all examined fungi, at highest concentration tested. *I. verum* showed the widest range of activities, resulting completely not effective (>19.52 mg/mL) against four *C. albicans* isolates and showing a MIC of 0.19 mg/mL versus two *C. famata* and one *C. albicans.* Results in detail of MICs expressed both as percentage and as mg/mL are reported in Table 5. No apparent relationship among the profiles of resistance to conventional drugs and MIC values of selected EOs was observed. 

## 3. Discussion

The present investigation reports the activity of selected EOs, against multidrug-resistant both bacterial and fungal organisms causing UTI in pet carnivores. At the best of our knowledge it is the first study that refers about drug-resistant veterinary isolates of *E. coli*, *Enterococcus* spp., *C. albicans* and *C. famata.*

The selected *E. coli* and *Enterococcus* spp. strains, previously isolated from dogs and cats with severe cases of UTI, resulted not sensitive (resistant and intermediate) to several antibiotics. In detail, one *E. coli* isolate was not sensitive to any tested antibiotic and some strains were sensitive only to one or two out of the twenty-one tested antibiotics.

The evaluation of the antibacterial activity of the selected EOs showed promising results, especially with *O. vulgare* and *T. vulgaris*. These EOs, in fact, resulted effective against all the tested isolates showing MIC values ranging from 0.15% (*v*/*v*) (0.293 mg/mL) to 0.6% (*v*/*v*) (1.183 mg/mL) for *O. vulgare*, and from 0.07% (*v*/*v*) (0.146 mg/mL) to 1.25% (*v*/*v*) (2.342 mg/mL) for *T. vulgaris*. These results are corroborated by previous investigations that found relevant antimicrobial properties of oregano and thyme EOs related to their main components carvacrol and thymol, but also other minor constituents such as the monoterpene hydrocarbons γ-terpinene and p-cymene [18]. *I. verum* and *S. sclarea* resulted moderately effective against *E. coli* strains, whereas no activity was observed when they were assayed versus *Enterococcus* spp. isolates. This different activity could be related to the dissimilar structure of Gram-positive and Gram-negative bacteria cell wall, as also supposed by Benmalek et al. [19] who found similar results when tested *I. verum* EO against *E. coli* and *Staphylococcus aureus*. Activity of *I. verum* against enterococci is scantly investigated; however, Hawrelak et al. [20] found a very weak effectiveness of this EO against *Enterococcus faecalis.* About *S. sclarea*, our results are comparable to those obtained by Frydrysiak et al. [21] who found weak antibacterial activity of *Salvia officinalis* and *Salvia lavandulaefolia*, whereas they are totally in disagreement with other studies in which strong activity of *S. officinalis* EO was observed against both Gram-negative bacteria, such as *E. coli*, and Gram-positive bacteria including enterococci [22]. This difference could be related to the original plant species and/or to variability among the bacterial strains. 

Basil EO showed a good antimicrobial activity, with MIC values ranging from 0.15% (*v*/*v*) (0.285 mg/mL) to 1.25% (*v*/*v*) (2.287 mg/mL), against all the selected *E. coli* strains, except for one isolate (n.986) that showed no-sensitivity to 19 antibiotics among the 21 tested. Very weak activity was observed against *Enterococcus* spp. isolates. Antibacterial activity of *O. basilicum* EO was demonstrated in other studies that assayed it against multi-drug resistant clinical isolates including *E. coli* [23] and *Enterococcus* [24]. Interestingly, *O. basilicum* EO chemical composition reported by Sienkiewicz et al. [23] differed considerably from the EO used in the present study being estragole the main represented compound (86.4%). Antimicrobial activity of *O. basilicum* EO is considered mainly related to eugenol that in our study is present in a moderate amount (11.5%); this could explain the lower MIC values found with respect to the other EOs.

Even though some among the tested oils did not show a very strong antimicrobial activity, the comparison to the standard drugs could be significant in some particular cases. For instance, the overall results obtained with *E. coli* strain n.876 are interesting, because this isolate was not-sensitive to all the tested antibiotics, but sensible to all the EOs, with low MIC values. These results suggest that there is no correlation between the sensitivity to conventional antibacterial drugs and to EOs. Such natural products could be an alternative when treating some clinical cases. 

Although mycotic cystitis is occasionally signaled in carnivores, causative agents investigated in the present study showed a wide range of resistance to commonly used antimycotic drugs. Fluconazole represents the preferred drug in the treatment of *Candida* UTI in human patients [25], while echinocandins and newer azoles are not routinely recommended, due to their low urinary concentrations [26]. Furthermore, itraconazole is the sole antimycotic drug allowed for systemic administration in veterinary medicine, and it is registered for treating feline microsporiasis, only. In this view, the local application of EOs formulations would be welcome. 

*T. vulgaris* appeared as the most effective EO, probably due to its high content of thymol (52.6%). This monoterpene compound, together with carvacrol, appeared to be able to completely block ergosterol synthesis at the MIC values [27], making porous the membrane and provoking the yeast cell killing. Carvacrol is mostly contained in *O. vulgare* EO (about 66%) that, in the present study, showed a good efficacy, although with slightly higher MICs when compared to *T. vulgaris*. Moreover, these compounds are reported as able to restore antifungal susceptibility to fluconazole in resistant *Candida* strains [28]. Eugenol is contained in moderate amounts (11.5%) in *O. basilicum* EO and shares with thymol the ability to damage cell membrane in *C. albicans* [29]. This EO resulted active against part of selected yeasts (2/4 *C. famata* and 5/12 *C. albicans*). Anethol is the main component (about 90%) of *I. verum* EO, and it is reported to have a weak antimicrobial action [30]. 

Antimicrobial activities of *I. verum* and its more represented component, anethol, have been extensively studied, mainly against phytopathogenic fungi [31,32,33,34], indicating a strong antifungal activity. Antifungal properties of *I. verum* EO and its main features have been extensively revised by Wang et al. [35], indicating a versatile use of this compound in ethnobotany. At the best of our knowledge, the only assays of this EO against zoopathogenic fungi are referred to dermatophytes [36] and to some agents of mycotic otitis in pets [37]. In both cases *I. verum* EO showed a poor activity. Moreover, in the present study it did not yield univocal results, showing wide differences in MIC values. The marked differences among the results obtained by us, would indicate a yeast individual sensitivity, considered that, for *C. albicans*, MIC were ranging from more than 10 to 0.1 mg/mL. For these reasons individual checking for the in vitro efficacy of EOs should be carried out prior to establish an alternative treatment.

An interesting finding would be the apparent absence of relation between the sensitivity to EOs with regards to the multidrug resistance.

Topical application of EOs based formulations has been reported in murine models of *Candida* vaginitis [38,39], indicating the feasibility of this route of administration. However, considered the wide variability of drug/EOs sensitivity patterns among the different isolates of *Candida*, a preliminary evaluation of the in vitro activity of selected compounds should be performed, to identify the most suitable EO to treat *Candida* UTI in pets. This assessment would be advisable, considering that other non-*albicans* species such as *C. famata*, *C. kefyr*, *C. inconspicua*, *C. rugosa*, *C. dubliniensis*, and *C. norvegensis*, although rarely isolated, are now considered emerging species, as their isolation rate has increased between 2- and 10-fold in human patients over the last 15 years [40].

## 4. Material and Methods

### 4.1. Essential Oils

EOs obtained from star anise (*I. verum* Hook.f.), basil (*O. basilicum* L.), origanum (*O. vulgare* L.), clary sage (*S. sclarea* L.) and thymus (*T. vulgaris* L.) were used in the present study. These EOs were selected for their antibacterial and antifungal actions reported in literature [35,41,42,43]. 

All EOs were purchased from the producer (FLORA^®^, Pisa, Italy) and maintained at 4 °C in dark glass vials until used. They were microbiologically analyzed for quality control before antibacterial and antimycotic tests.

### 4.2. Gas Chromatography—Mass Spectrometry Analysis 

The GC analysis was performed as previously described [44]. 

### 4.3. Antibacterial Activity

#### 4.3.1. Bacterial Strains

Seven *E. coli* and eight *Enterococcus* spp. strains were employed in the study. The strains were previously isolated from female dogs and cats with urinary tract infections. *E. coli* and *Enterococcus* spp. strains were typed using API 20E and API 20STREP System (BioMérieux, Marcy l’Etoile, France), respectively and stored in glycerol broth at −80 °C. 

#### 4.3.2. Agar Disk Diffusion Method

The in vitro sensitivity of each *E. coli* and *Enterococcus* spp. strain to the following antibiotics (Oxoid Ltd. Basingstoke, Hampshire, UK) was tested: aztreonam (30 µg), amikacin (30 µg), amoxycillin-clavulanic acid (30 μg), ampicillin (10 µg), cephalothin (30 µg), cefotaxime (30 µg), ceftazidime (30 µg), cephalexin (30 µg), ciprofloxacin (5 µg), colistin sulfate (10 µg), doxycycline (30 µg), erythromycin (10 µg), enrofloxacin (5 µg), gentamicin (10 µg), neomycin (30 µg), piperacillin (100 µg), rifampicin (30 µg), streptomycin (10 µg), sulphametoxazole-trimethoprim (25 µg), tetracycline (30 µg), tobramycin (10 µg). The in vitro sensitivity to the antibiotics was evaluated by Kirby-Bauer agar disk diffusion method and the results were interpreted as indicated by the National Committee for Clinical Laboratory Standards (NCCLS) [45]. 

Antibacterial activity of each EO was tested by Kirby-Bauer agar disk diffusion method following the procedures reported by Clinical and Laboratory Standards Institute (CLSI) [46] with some modifications. In details, each EO and mixture was diluted 1:10 in dimethyl sulfoxide (DMSO, Oxoid Ltd.) and one absorbent paper disk was impregnated with 10 µL of each dilution, respectively. A paper disk impregnated with 10 µL of DMSO was included as negative control. All tests were performed in triplicate.

#### 4.3.3. Minimum Inhibitory Concentration 

Minimum inhibitory concentration (MIC) was determined with agar disk diffusion method and broth microdilution assay. For agar disk diffusion method 10 µL of 10%, 5%, 2.5%, 1.25%, 0.62%, 0.31%, 0.15%, 0.07%, 0.03% (*v*/*v*) of each EO in DMSO were added on paper disks. 

Microdilution assay was performed in 96-well microtitre plates following the protocol previously described [43]. Briefly, the test was carried out in a total volume of 200 µL including 160 µL of brain hearth infusion broth (BHIB, Oxoid Ltd.), 20 µL of each bacterial suspension and 20 µL of each oil with final EOs concentrations ranging from 10% to 0.03%. Plates were incubated at 37 °C for 24 h. The same assay was performed simultaneously for bacterial growth control (tested bacteria and BHIB) and sterility control (tested oil or mixture and BHIB). All tests were executed in triplicate. The MIC value was defined as the lowest concentration, expressed as mg/mL, of EO at which microorganisms show no visible growth.

### 4.4. Antimycotic Activity

#### 4.4.1. Fungal Strains

Four strains of *C. famata* and twelve of *C. albicans* were used for the assays. All the yeasts were clinical isolates from female dogs. Identification was achieved using physiological tests such as cultivation onto Corn Meal Agar (Sigma Aldrich, Milano, Italy) and germ-tube. Microscopy and biochemical profile evaluated by ID 32 (BioMérieux), were performed, also. When a doubtful profile was obtained, a final identification was carried out by molecular methods. 

#### 4.4.2. Minimal Inhibitory Concentration

Selected EOs were tested by a microdilution assay, as recommended by CLSI M27A_3_ for yeasts [47], using dilutions of 10%, 5%, 2.5%, 2%, 1.5%, 1%, 0.75%, 0.5%, 0.25%, 0.2%, 0.1%, 0.075%, 0.05%, 0.025%, 0.01% and 0.005% to achieve a MIC value. Sensitivity to conventional antimycotic drugs (fluconazole, voriconazole, itraconazole and caspofungin) was evaluated by Etest (BioMérieux) and breakpoint values, when available, were calculated following the CLSI recommendations for *Candida* spp. [48].

## 5. Conclusions

UTIs are frequent reason for antimicrobial treatment in dogs and cats. Choice of antibiotics and/or antifungal products should follow the in vitro evaluation of causative agent sensitivity. However, sometimes these microorganisms persist in the urogenital tract and/or a new infection can occur. The use of EOs has been proposed for the treatment of human UTIs, so these natural products could be evaluated in veterinary medicine, also, mainly when clinical healing cannot be achieved by using conventional drugs. Moreover, EOs could be used for relapses prevention. An in vitro evaluation of the isolated pathogens sensitivity to different EOs should be performed to select an oil for UTI treatment. As reported in literature for other microorganisms, in the present study *T. vulgaris* and *O. vulgare* EOs showed the strongest antimicrobial activity against *E. coli*, *Enterococcus* spp., *C. albicans* and *C. famata*, so they could be proposed for the formulation of external and/or intravesical washes, after a careful evaluation of both cytotoxicity and therapeutic index.

## Figures and Tables

**Table 1:** Relative percentage of the main constituents of tested essential oils.

	Class	Component	RI	*O.v*	*O.b*	*S.s*	*T.v*	*I.v*
2	mh	tricyclene	926					1.4
9	mh	myrcene	991	2.2				
13	mh	*α*-terpinene	1018	2.1				
15	mh	p-cymene	1026	9.3			15.3	
16	mh	limonene	1031					3.9
18	om	1,8-cineole	1033		5.9			
22	mh	*γ*-terpinene	1062	5.3			2.9	
25	om	*cis*-linalool oxide (furanoid)	1074			2.2		
28	om	*trans*-linalool oxide (furanoid)	1088			1.8		
30	om	*trans*-sabinene idrato	1097	1.8			3.8	
31	om	linalool	1098		46	8.1		
39	om	borneol	1165				1.6	
41	om	4-terpineol	1177				2.4	
43		unknown					1.7	
44	pp	menthyl chavicol(=estragole)	1195		1.1			
46	om	thymol methyl ether	1232				1.7	
49	om	linalyl acetate	1257			54.7		
53	pp	(E) anethol	1283					89.8
54	om	isobornyl acetate	1285		1.6			
56	om	thymol	1290				52.6	
58	om	carvacrol	1298	65.9				
59		unknown				5.6		
60		unknown				7.2		
61	om	*α*-limonene diepoxide	1347			8.6		
62	pp	eugenol	1356		11.5			
65	sh	*β*-elemene	1392		2.2			
66	sh	*β*-caryophyllene	1418	3.7			6.8	
67	sh	*trans*-*α*-bergamotene	1437		3.6			
69	sh	*α*-guaiene	1440		1.1			
72	sh	germacrene D	1481		3.5			
74	sh	*α*-bulnesene	1505		2			
75	sh	*trans*-*γ*-cadinene	1513		2.8			
77	sh	*δ*-cadinene	1524				1	
80	os	caryophyllene oxide	1581			4.8		
82	os	1,10-di-epi-cubenol	1614		1			
83	os	T-cadinol	1640		5.8			
87	od	scareol	2223			1.3		

Legend: RI: retention index measured on HP-5 column; *O.v.*: *Origanum vulgare*; *O.b.*: *Ocimum basilicum*; *S.s.*: *Salvia sclarea*; *T.v.*: *Thymus vulgaris*; *I.v.*: *Illicium verum*; mh: monoterpene hydrocarbons; om: oxygenated monoterpenes; sh: sesquiterpene hydrocarbons; os: oxygenated sesquiterpenes; pp: phenylpropanoids; od: oxygenated diterpenes.

**Table 2:** Results of the agar disc diffusion method testing each *Escherichia coli* and *Enterococcus* spp. isolates with different antibiotics.

Antibiotic	*Escherichia coli* Strain n°							*Enterococcus* spp. Strain n° *							
	1069	1002	994	986	977	876	835	1091	1079	1034	835	793	654	618	568
ATM	R	R	R	I	R	R	S	R	R	R	R	R	R	R	R
AK	S	I	S	R	S	R	S	R	R	R	R	R	I	S	R
AMC	R	I	R	I	I	R	R	S	S	S	S	S	S	S	S
AMP	R	R	R	R	R	R	R	R	R	R	I	R	I	I	I
KF	R	R	R	R	R	R	R	R	R	R	R	I	I	S	S
CTX	I	R	R	S	R	I	S	R	R	R	I	R	R	R	I
CAZ	R	R	R	R	R	R	S	R	R	R	R	R	R	R	R
CL	R	R	R	R	R	I	R	R	R	R	R	R	R	I	R
CIP	R	I	R	R	R	R	R	R	R	R	R	R	S	R	R
CT	R	S	S	R	S	R	S	R	R	R	R	R	R	R	R
DO	R	S	S	R	R	R	R	R	S	I	R	R	S	S	R
E	R	R	R	R	R	R	R	I	I	R	R	R	I	S	R
ENR	R	S	R	R	R	R	R	R	R	R	R	R	I	R	R
CN	R	S	S	S	S	R	R	R	R	I	R	I	S	S	R
N	R	I	I	R	R	R	I	R	R	R	R	R	I	I	R
PPL	R	R	R	R	R	R	R	I	I	R	I	R	I	R	I
RD	I	R	R	I	I	R	I	R	R	I	S	R	S	I	S
S	R	I	I	I	I	R	R	R	R	R	R	R	R	I	R
STX	R	S	S	R	R	R	R	S	R	S	S	S	S	S	R
TE	R	S	S	R	R	R	R	R	S	R	R	R	R	R	R
TOB	R	I	S	R	R	R	R	R	R	R	R	R	S	S	R

Legend—S: sensitive; I: intermediate; R: resistant; ATM: aztreonam; AK: amikacin; AMC: amoxicillin-clavulanic acid; AMP: ampicillin; KF: cephalotin; CTX: cefotaxime; CAZ: ceftazidime; CL: cephalexin; CIP: ciprofloxacin; CT: colistin sulfate; DO: doxycycline; E: erythromycin; ENR: enrofloxacin; CN: gentamicin; N: neomycin; PPL: piperacillin; RD: rifampicin; S: streptomycin; SXT: sulphametoxazole-trimethoprim; TE: tetracycline; TOB: tobramycin; *: *Enterococcus faecium* (strains 1091 ,1079, 1034), *Enterococcus faecalis* (strains 835, 793, 654, 618), *Enterococcus durans* (strain 568).

**Table 3:** Number and percentages of *Escherichia coli* and *Enterococcus* spp. isolates resulted sensitive, intermediate or resistant versus tested antibiotics.

Antibiotic	*Escherichia coli*			*Enterococcus* spp*.*		
	S (%)	I (%)	R (%)	S (%)	I (%)	R (%)
ATM	1 (14.3)	1 (14.3)	5 (71.4)	0	0	8 (100)
AK	4 (57.1)	1 (14.3)	2 (28.6)	1 (12.5)	1 (12.5)	6 (75)
AMC	0	3 (42.9)	4 (57.1)	8 (100)	0	0
AMP	0	0	7 (100)	0	4 (50)	4 (50)
KF	0	0	7 (100)	2 (25)	2 (25)	4 (50)
CTX	2 (28.6)	2 (28.6)	3 (42.8)	0	2 (25)	6 (75)
CAZ	1 (14.3)	0	6 (85.7)	0	0	8 (100)
CL	0	1 (14.3)	6 (85.7)	0	1 (12.5)	7 (87.5)
CIP	0	1 (14.3)	6 (85.7)	1 (12.5)	0	7 (87.5)
CT	4 (57.1)	0	3 (42.9)	0	0	8 (100)
DO	2 (28.6)	0	5 (71.4)	3 (37.5)	1 (12.5)	4 (50)
E	0	0	7 (100)	1 (12.5)	3 (37.5)	4 (50)
ENR	1 (14.3)	0	6 (85.7)	0	1 (12.5)	7 (87.5)
CN	4 (57.1)	0	3 (42.9)	2 (25)	2 (25)	4 (50)
N	0	4 (57.1)	3 (42.9)	0	2 (25)	6 (75)
PPL	0	0	7 (100)	0	5 (62.5)	3 (37.5)
RD	0	3 (42.9)	4 (57.1)	3 (37.5)	2 (25)	3 (37.5)
S	0	4 (57.1)	3 (42.9)	0	1 (12.5)	7 (87.5)
STX	2 (28.6)	0	5 (71.4)	6 (75)	0	2 (25)
TE	2 (28.6)	0	5 (71.4)	1 (12.5)	0	7 (87.5)
TOB	0	2 (28.6)	5 (71.4)	2 (25)	0	6 (75)

Legend—S: sensitive; I: intermediate; R: resistant; ATM: aztreonam; AK: amikacin; AMC: amoxicillin-clavulanic acid; AMP: ampicillin; KF: cephalotin; CTX: cefotaxime; CAZ: ceftazidime; CL: cephalexin; CIP: ciprofloxacin; CT: colistin sulfate; DO: doxycycline; E: erythromycin; ENR: enrofloxacin; CN: gentamicin; N: neomycin; PPL: piperacillin; RD: rifampicin; S: streptomycin; SXT: sulphametoxazole-trimethoprim; TE: tetracycline; TOB: tobramycin.

**Table 4:** Antibacterial activity expressed as minimum inhibitory concentration (MIC) of the essential oils against *Escherichia coli* and *Enterococcus* strains *.

Bacterial Strain	*Illicium verum*		*Ocimum basilicum*		*Origanum vulgare*		*Salvia sclarea*		*Thymus vulgaris*	
	%	mg/mL	%	mg/mL	%	mg/mL	%	mg/mL	%	mg/mL
*E. coli* 1069	0.3	0.611	0.3	0.571	0.15	0.293	0.15	0.279	0.3	0.585
*E. coli* 1002	0.3 0.611		1.25	2.287	0.6	1.183	1.25	2.232	0.3	0.585
*E. coli* 994	0.07	0.152	0.15	0.285	0.15	0.293	0.15	0.279	0.07	0.146
*E. coli* 986	1.25	2.445	NE		0.3	0.587	1.25	2.232	0.3	0.585
*E. coli* 977	1.25	2.445	1.25	2.287	0.3	0.587	1.25	2.232	0.15	0.292
*E. coli* 876	0.07	0.152	0.6	1.143	0.15	0.293	0.07	0.139	0.07	0.146
*E. coli* 835	0.3	0.611	0.6	1.143	0.3	0.587	0.3	0.558	0.07	0.146
*Enterococcus* 1091	NE		10	18.3	0.6	1.183	NE		1.25	2.342
*Enterococcus* 1079	NE		2.5	4.575	0.6	1.183	NE		1.25	2.342
*Enterococcus* 1034	NE		5	9.15	0.6	1.183	NE		0.6	1.171
*Enterococcus* 835	NE		5	9.15	0.6	1.183	NE		1.25	2.342
*Enterococcus* 793	NE		1.25	2.287	0.6	1.183	NE		1.25	2.342
*Enterococcus* 654	NE		1.25	2.287	0.6	1.183	NE		1.25	2.342
*Enterococcus* 618	NE		2.5	4.575	0.6	1.183	NE		1.25	2.342
*Enterococcus* 568	NE		5	9.15	0.6	1.183	NE		0.6	1.171

Legend—NE: no effective; *: *Enterococcus faecium* (strains 1091, 1079, 1034), *Enterococcus faecalis* (strains 835, 793, 654, 618), *Enterococcus durans* (strain 568).

**Table 5:** MIC values of selected essential oils, expressed as percentage and weights.

Yeast Strain	*Ocimum basilicum* %mg/mL		*Origanum vulgare* %mg/mL		*Salvia sclarea* %mg/mL		*Thymus vulgaris* %mg/mL		*Illicium verum* %mg/mL		Caspofungin * mg/L	Voriconazole ° mg/L	Itraconazole mg/L	Fluconazole § mg/L
*C famata1*	0.1	0.18	0.1	0.18	>10	>17.86	0.075	0.14	0.1	0.19	0.125	0.25	0.25	2
*C famata2*	2.5	4.58	1.25	2.25	>10	>17.86	0.075	0.14	1.5	2.93	0.047	0.25	1	16
*C famata3*	1.5	2.7	1.25	2.25	>10	>17.86	0.075	0.14	1	1.95	0.047	1	1	32
*C famata4*	0.075	0.13	0.075	0.135	>10	>17.86	0.075	0.14	0.1	0.19	0.125	0.125	1	8
*C.albicans1*	0.075	0.13	0.075	0.135	>10	>17.86	0.05	0.09	1	1.95	0.125	>32	>32	>256
*C.albicans2*	0.5	0.9	2	3.6	>10	>17.86	0.5	0.93	>10	>19.56	0.125	>32	>32	>256
*C.albicans3*	0.075	0.13	0.075	0.135	>10	>17.86	0.075	0.14	0.1	0.19	0.125	>32	>32	>256
*C.albicans4*	1	1.8	1	1.8	>10	>17.86	0.1	0.19	1.25	2.44	0.125	>32	>32	2
*C.albicans5*	0.5	0.9	0.1	0.18	>10	>17.86	0.1	0.19	>10	>19.56	0.125	>32	>32	>256
*C.albicans6*	2.5	4.58	1	1.8	>10	>17.86	0.1	0.19	2	3.9	0.125	>32	>32	>256
*C.albicans7*	1	1.8	0.075	0.135	>10	>17.86	1	1.87	2	3.9	0.125	>32	>32	0.75
*C.albicans8*	0.75	1.3	0.1	0.18	>10	>17.86	0.075	0.14	2	3.9	0.094	>32	>32	>256
*C.albicans9*	0.05	0.09	0.01	0.018	>10	>17.86	0.05	0.09	1	1.95	0.19	>32	>32	>256
*C.albicans10*	1	1.8	0.75	1.35	>10	>17.86	0.5	0.93	0.5	0.97	0.125	>32	>32	2
*C.albicans11*	2.5	4.58	0.05	0.09	>10	>17.86	0.05	0.09	>10	>19.56	0.19	>32	>32	>256
*C.albicans12*	1	1.8	0.1	0.18	>10	>17.86	0.1	0.19	>10	>19.56	0.125	1	>32	>256

Legend—* breakpoint of echinocandins recommended by CLSI for *C. albicans* up to 0.25 (sensitive), 0.5 (intermediate), from 1 (resistant); ° recommended breakpoint by CLSI for *C. albicans* up to 0.12 (sensitive), 0.25–0.5 (intermediate), from 1 (resistant); § recommended breakpoint by CLSI for *C. albicans* up to 2 (sensitive), from 8 (resistant).
